# Association between neighborhood health behaviors and body mass index in Northern Norway: evidence from the Tromsø Study

**DOI:** 10.1177/14034948211059972

**Published:** 2021-12-13

**Authors:** Emre Sari, Mikko Moilanen, Clare Bambra, Sameline Grimsgaard, Inger Njølstad

**Affiliations:** 1 UiT the Arctic University of Norway; 2 Vrije University Amsterdam; 3 Faculty of Medical Sciences, Newcastle University, UK; 4 Department of Community Medicine, UiT The Arctic University of Norway, Norway

**Keywords:** Obesity, overweight, body mass index, health behavior, residence characteristics, leisure activities, exercise, health risk behaviors, Norway, longitudinal studies

## Abstract

**Aim:**

The prevalence of overweight and obesity has risen rapidly worldwide, and the ongoing obesity pandemic is one of the most severe public health concerns in modern society. The average body mass index (BMI) of people living in Northern Norway has also steadily increased since the late 1970s. This study aimed to understand how individuals’ health behavior is associated with the general health behavior of the people in their neighborhood.

**Methods:**

Using the population-based Tromsø Study, we examined the life course association between average leisure time physical activity at the neighborhood level and the BMI of individuals living in the same neighborhood. We used a longitudinal dataset following 25,604 individuals living in 33 neighborhoods and performed a linear mixed-effects analysis.

**Results:**

The results showed that participants living in neighborhoods whose residents were more physically active during their leisure time, were likely to have a significantly lower BMI (−0.9 kg/m², 95% CI −1.5 to −0.4). Also, individuals living in neighborhoods whose residents were doing mainly manual work, had significantly higher BMIs (0.7 kg/m², 95% CI 0.4−1.0).

**Conclusions:**

**Our results showed a strong association between the average leisure time physical activity level of neighborhood residents and the higher BMI levels of residents of the same neighborhood.**

## Background

Overweight and obesity are on the rise across the world, and their combined effect has emerged as one of the most severe public health issues facing modern society. Obesity is known to have a complex pathology, with evidence of numerous underlying causes. One of many factors that influences the pathway to obesity is the relationship between a neighborhood’s socioeconomic conditions and its residents’ risky health behaviors [1].

The imbalance between calorie intake and burning formed by behavioral risk factors such as a poor diet, a sedentary lifestyle, or unhealthy habits is one of the primary causes of a high body mass index (BMI) for both overweight (25 kg/m² ⩾ BMI < 30 kg/m²) and obesity (BMI ⩾ 30 kg/m²) [2]. Overweight and obesity are important risk factors of non-communicable diseases such as diabetes, musculoskeletal disorders, obstructive sleep apnea, and some types of cancer (prostate, colorectal, endometrial, and breast) [3].

The mechanisms underlying BMI have been explored in various ways in economics, epidemiology, sociology, medicine, and geography [4]. The effect of a neighborhood’s physical and social structure on the lives of the residing individuals is considered to be one of these mechanisms. In the literature, neighborhood effects refer to various neighborhood circumstances, such as the characteristics of individuals living in the same areas that influence residents’ well-being [5]. Galster categorizes the “neighborhood effect” theory broadly under four mechanisms: social interaction, environmental, geographical, and institutional [6]. In another major study, Durlauf emphasizes that both role models and peer group influences often produce imitation behavior contemporaneously or across age groups [7]. In this study, we concentrated on social-interactive mechanisms.

We studied whether the higher BMI levels of neighborhood residents were associated with the average leisure time physical activity level of the same neighborhood residents. Several studies, using longitudinal data provide important evidence that the socioeconomic and physical characteristics of the environment in which individuals live influence their BMIs [8−11]. Among these, one of the studies for Norway is Sund et al.’s study [11]. They use the two waves of the Nord-Trøndelag Health Study to examine relationships between area, family, and individual characteristics with BMI and BMI change. However, to our knowledge, no longitudinal studies have previously been used to investigate the relationship between overall neighborhood health behaviors and individual-level BMI.

Our study, therefore, makes a contribution to the international literature by longitudinally examining how the average leisure time physical activity of residents in a neighborhood is associated with the BMI of individuals living in that neighborhood.

## Methods

### The Tromsø Study

The Tromsø Study is a cohort study involving residents of the municipality of Tromsø, which is the largest city in Northern Norway, with around 77,000 inhabitants. The study was first initiated in 1974 to help reduce the high cardiovascular disease (CVD)-caused mortality rates in Norway. In addition to identifying causes of high CVD mortality and supporting CVD prevention, it has also focused on other chronic diseases and conditions. In general, the Tromsø Study has comprises the following: demographics; questionnaire; interview; physical examinations such as measured weight, and height,  blood pressure, and various types of blood samples; and some other clinical examinations [12]. The Tromsø Study was performed in seven waves (referred to as Tromsø 1−7) from 1974 to 2016 and had a participation rate ranging from 64.7−78.5%. It was funded by UiT The Arctic University of Norway [12].

**Figure 1.**
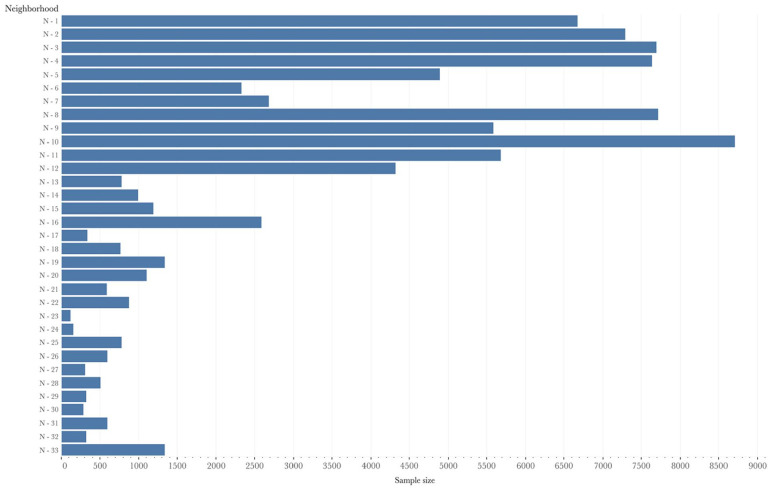
Total sample size of each neighborhood. Depending on the neighborhood, the distribution of our total sample varies. The sample sizes are presented on the x-axis, and the neighborhoods are shown on the y-axis.

In our study, we used six waves of the Tromsø Study: Tromsø 2 in 1979/80 (*N* = 16,621; age group: 20−54), Tromsø 3 in 1986/87 (*N* = 21,826; age group: 12−67), Tromsø 4 in 1994/95 (*N* = 27,158; age group: 25−97), Tromsø 5 in 2001/02 (*N* = 8,130; age group: 30−89), Tromsø 6 in 2007/08 (*N* = 12,984; age group: 30−87), and Tromsø 7 completed in 2015/16 (*N* = 21,083; age group: 40 and older) [12]. We generated longitudinal data by tracing individuals who had participated in at least two waves of the Tromsø Study. In the final dataset, we included 25,604 unique individuals residing in 33 different constituencies. To define the neighborhoods in our article, we used constituency-level information for each individual [11]. In Figure 1, we present the total sample size of each neighborhood.

### Variables

#### Outcome

Our health outcome was defined as an individual’s BMI [1,4,9,11]. The BMI is one method used to identify overweight and obesity in individuals [13]. The BMI of each individual in our study was calculated by generating a function of the participants’ weight in kilograms divided by the square of his or her height in meters (kg/m^2^ ). According to the World Health Organization (WHO), adults with BMI values less than 18.5 kg/m^2^ are classified as underweight; adults with BMI values between 18.5 and 24.9 are classified as normal weight; adults with BMI values between 25 and 29.9 are classified as overweight, and adults with a BMI above 30 are classified as obese.

#### Individual-level explanatory variables

We used the levels of leisure time physical activity reported by the Tromsø Study participants to classify sedentary health behavior [1,11,14]. The question was, “Describe your exercise and physical exertion in leisure time over the last year. If your activity varies throughout the year, give an average.” The answers were grouped under four levels: (a) reading, watching TV/screen, or other sedentary activity (reference category); (b) an activity that requires walking or cycling; (c) participation in recreational sports; (d) participation in hard training or sports competitions.

Additionally, we included individual-level demographic, socioeconomic status (SES), and health behavior variables for Tromsø 2−7 (see Table I). Age and age squared were modeled as continuous variables, with both centered on their mean in years [11]. Gender was modeled as a dummy variable [8], and women were used as the reference category. Marital status was classified into three groups: unmarried (reference category), married/registered partnership, and divorced/widow(er)/separated. The SES variables were self-reported and based on both the participants’ education level and occupational status as well as their mothers’ educational level. We divided the education variables into high (completed 4 years or more at a college/university degree), medium (having a high/technical school diploma), and low (completed an elementary school or no education) as a reference category [8]. Additionally, in order to see the effect of working requirements, we used occupational status like Sund et al. [11] and considered occupational status variables, that is, unemployed/retired, blue-collar, and white-collar occupations, as proxy measures for physical activity levels during work. The question asked in the surveys was, “If you have paid or unpaid work, which statement describes your work best?” and the answers were arranged in four levels from entirely sedentary work (white-collar worker) to heavy manual labor (blue-collar worker). It is generally accepted that white-collar work requires more mental and less physical effort than blue-collar work [15].

**Table I.** Sample Characteristics.

	**Tromsø 2** (1979−80)		**Tromsø 3** (1986−87)		**Tromsø 4** (1994−95)		**Tromsø 5** (2001−02)		**Tromsø 6** (2007−08)		**Tromsø 7** (2015−16)	
	***N***	**Mean (%)**	***N***	**Mean (%)**	***N***	**Mean (%)**	***N***	**Mean (%)**	***N***	**Mean (%)**	***N***	**Mean (%)**
*Individual level*												
Body mass index	12,985	23.51 (3.19)	17,952	23.96 (3.35)	21,113	25.18 (3.77)	7690	26.61 (4.24)	12,133	26.90 (4.26)	15,143	27.34 (4.45)
Age (years)	12,985	34.97 (8.80)	17,952	38.53 (10.43)	21,113	45.88 (12.35)	7690	60.52 (13.35)	12,133	58.23 (12.30)	15,143	61.15 (10.22)
Sex												
Females (ref)	6379	49.1	8951	49.9	10,999	52.1	4384	57.0	6496	53.5	8071	53.3
Males	6606	50.9	9001	50.1	10114	47.9	3306	43.0	5637	46.5	7072	46.7
Marital Status												
Unmarried (ref)	3303	25.4	5252	29.3	5272	25.0	1086	14.1	2011	16.6	2798	18.5
Married/registered partnership	8950	68.9	11,022	61.4	12,501	59.2	4772	62.1	7310	60.2	8950	59.1
Divorced/widow(-er)/separated	732	5.6	1677	9.3	3340	15.8	1832	23.8	2812	23.2	3395	22.4
Education												
Low (ref)	6619	58.5	8105	48.9	7083	33.7	4210	57.3	3525	29.5	4094	27.6
Moderate	3947	34.9	6885	41.5	7887	37.5	2350	32.0	4039	33.8	7049	47.6
High	752	6.6	1593	9.6	6072	28.9	787	10.7	4402	36.8	3678	24.8
Mothers’ education												
Low (ref)	7067	87.7	9843	84.4	12,130	82.1	4461	85.3	9266	80.1	11,642	78.2
Moderate	840	10.4	1529	13.1	2194	14.9	629	12.0	1715	14.8	2629	17.7
High	147	1.8	288	2.5	449	3.0	137	2.6	583	5.0	616	4.1
Occupation												
Unemployed or retired (ref)	394	3.0	583	3.2	700	3.3	60	0.8	52	0.4	1493	9.9
White-collar worker	4289	33.0	6916	38.5	7107	33.7	1966	25.6	4084	33.7	5852	38.6
Blue-collar worker	8302	63.9	10,453	58.2	13,306	63.0	5664	73.7	7997	65.9	7798	51.5
Physical activity at leisure times												
Mostly sedentary activity (ref)	2679	20.7	4140	23.1	N/A	N/A	1025	19.7	2252	20.2	2050	14.1
Activity that requires walking or cycling	7209	55.6	10,492	58.5	N/A	N/A	3402	65.5	6674	60.0	8791	60.4
Participation in recreational sports	2656	20.5	2865	16.0	N/A	N/A	694	13.4	2023	18.2	3358	23.1
Participation in hard training or sports competitions	427	3.3	447	2.5	N/A	N/A	70	1.3	173	1.6	351	2.4
*Neighborhood level*												
Physical Activity at leisure times	12,985	2.06 (0.06)	17,952	2.02 (0.06)	N/A	N/A	7690	1.97 (0.06)	12,133	2.01 (0.06)	15,143	2.16 (0.07)
Physical activity at work	12,985	1.98 (0.23)	17,952	1.89 (0.20)	21,113	1.93 (0.25)	7690	1.80 (0.23)	12,133	1.73 (0.22)	15,143	1.66 (0.18)
Type of neighborhood												
Urban (ref)	10,792	83.1	12,698	70.7	18,376	87.0	6692	87.0	10,776	88.8	12,181	80.4
Rural	2193	16.9	5254	29.3	2737	13.0	998	13.0	1357	11.2	2962	19.6

Note: BMI classification is as follows: BMI < 18.5 kg/m² is underweight, 18.5 kg/m² ⩾ BMI < 25 kg/m² is normal weight, 25 kg/m² ⩾ BMI < 30 kg/m² is overweight, 30 kg/m² ⩾ BMI is obesity. Education level is classified under three levels: low education postulates 7−10 years primary/secondary school and modern secondary school; moderate education covers high school and technical school diploma; and high education represents college/university or more. Urban and rural differentiation was done by using the classification in Hopstock et al.[17] Neighborhood-level variables were manually generated by the average results of individuals who lived in the same neighborhood.

#### Neighborhood-level explanatory variables

With the intention to investigate how neighborhood health behavior is associated with individual health (represented here by BMI), this study focused on interactive social mechanisms by using neighborhood-level leisure time physical activity as neighborhood-level health behavior. We refer to social contagion, collective socialization, and social networks as endogenous social processes within the interactive social mechanisms. However, we examined the average physical activity level of neighborhood residents at work and respondents’ urban−rural segregation with a view to controlling institutional and geographical mechanisms, respectively (for an extensive explanation of mechanisms, see Galster [6]). Neighborhood-level leisure time physical activity and physical activity at work were derived by averaging the individual participant responses for each neighborhood in Tromsø 2−7 [16].

#### Statistical analyses

We performed a linear mixed-effects (LME) analysis of the relationship between individual BMI and the average leisure time physical activity level of the neighborhood [8,11]. The LME models distinguished the individual- and neighborhood-level sources of variation in BMI and facilitated simultaneous examination of their effects separately. Additionally, for longitudinal data, LME allowed us to detect both variations between individuals and inter-group differences (variations among higher-level units), as well as estimates of change for single individuals. The fundamental and methodological importance of LME models has been defined comprehensively in literature [11,18,19].

In our study, we estimated two-level models with the continuous responses of BMI for individuals (Level 1) nested within neighborhoods (Level 2). We used restricted maximum likelihood to estimate the model parameters [18], and fitted them by using the lme4 v1.1-25 package [20]. By following Sund et al. [11], first, we developed a model (Model A/1) that was fitted with a null (empty) model to determine the variation in outcome between the two levels. Second, we included individual-level demographics (age, age squared, gender, and marital status) and the year of each Tromsø Study as control (Model A/2). Third, we included SES variables (Model A/3): education, mother’s education, and occupation. Fourth, we entered leisure time physical activity level of the neighborhood residents (Model A/4). Lastly, in addition to individual-level control variables, we entered neighborhood-level variables (Model A): mean physical activity level during leisure time, mean physical activity level at work, and the urban−rural divide. In addition, we separated the models into two categories: fixed effects and random effects. Fixed effects are predictor variables whose effects do not vary between individuals and neighborhoods. The variance in outcome at the individual and neighborhood levels that remains after controlling for the fixed effects are the random effects [11]. Finally, we developed a second model (Model B), a subgroup of Model A, which included only individuals observed in the same neighborhood throughout the Tromsø Study waves to control our results’ robustness.

LME models are flexible enough to deal with unbalanced data [21], such as the data we used in this study. However, to be on the safe side, we tested our model’s outputs with Kenward−Roger’s *F* test to assess the significance of the fixed effects [22], and found the same significance levels for the same variables. We also conducted the variance inflation factor (VIF) analysis to check the probability of multicollinearity risk in our models. The results of the VIF analysis varied from 1.019 to a maximum of 3.416; the only exceptions were age, age squared, and time, as expected. Therefore, we determined that multicollinearity was not a risk factor in our models since the results were close to the smallest possible value for VIF [23]. Finally, we used the intraclass correlation coefficient (ICC) to report variations among higher-level units. In the model, we determined with ICC the percentage of total unexplained variance for each level. The results are presented in Table II below.

**Table II.** The predictors of BMI.

Coefficient	**Model A/1**		**Model A/2**		**Model A/3**		**Model A/4**		**Model A**		**Model B**	
	Estimates	CI (95%)	Estimates	CI (95%)	Estimates	CI (95%)	Estimates	CI (95%)	Estimates	CI (95%)	Estimates	CI (95%)
*Fixed effects*												
Individual level (intercept)	25.545***	25.134−25.956	24.176***	24.045−24.308	24.156***	23.975−24.338	24.536***	24.320−24.751	24.782***	23.733−25.831	26.584***	24.370−28.798
Demography												
Age (years)			0.231***	0.224−0.239	0.208***	0.199−0.218	0.204***	0.192−0.215	0.205***	0.193−0.216	0.207***	0.183−0.231
Age (years)^2^			−0.002***	−0.002 to −0.002	−0.002***	−0.002 to −0.002	-0.002***	−0.002 to −0.002	−0.002***	−0.002 to −0.002	−0.002***	−0.002 to −0.002
Gender (male)			0.966***	0.880−1.053	1.066***	0.963−1.169	1.152***	1.046−1.258	1.147***	1.040−1.253	0.968***	0.831−1.106
Marital status												
Married/registered partnership			−0.219***	−0.282 to −0.156	−0.220***	−0.292 to −0.147	−0.239***	−0.328 to −0.150	−0.238***	−0.326 to −0.149	−0.255***	−0.412 to −0.098
Divorced/widow(-er)/separated			−0.306***	−0.385 to −0.228	−0.318***	−0.409 to −0.226	−0.265***	−0.377 to −0.152	−0.260***	−0.373 to −0.148	−0.193*	−0.390−0.003
Waves												
Tromsø Study 3 (1986−87)			0.150***	0.101−0.200	0.245***	0.178−0.312	0.257***	0.185−0.329	0.296***	0.218−0.373	0.225**	0.033−0.417
Tromsø Study 4 (1994−95)			0.840***	0.771−0.910	1.084***	0.989−1.178						
Tromsø Study 5 (2001−02)			1.692***	1.594−1.790	2.099***	1.969−2.229	2.184***	2.041−2.327	2.266***	2.115−2.418	2.090***	1.793−2.387
Tromsø Study 6 (2007−08)			1.985***	1.875−2.094	2.391***	2.242−2.541	2.569***	2.406−2.731	2.735***	2.565−2.904	2.826***	2.536−3.117
Tromsø Study 7 (2015−16)			2.409***	2.276−2.542	2.803***	2.622−2.984	2.999***	2.806−3.192	3.353***	3.148−3.557	3.569***	3.248−3.890
SES												
Education												
Moderate					−0.101***	−0.159 to −0.043	−0.127**	−0.199 to −0.055	−0.113**	−0.185 to −0.041	−0.138**	−0.272 to −0.004
High					−0.292***	−0.373 to −0.211	−0.440***	−0.542 to −0.339	−0.419***	−0.520 to −0.317	−0.672***	−0.849 to −0.496
Mother’s education												
Moderate					−0.334***	−0.477 to −0.191	−0.279***	−0.427 to −0.132	−0.264***	−0.412 to −0.117	−0.195**	−0.382 to −0.008
High					−0.593***	−0.862 to −0.323	−0.547***	−0.824 to −0.271	−0.530***	−0.807 to −0.254	−0.390**	−0.738 to −0.042
Occupation												
White-collar worker					−0.059	−0.152−0.035	−0.114*	−0.232−0.004	−0.100*	−0.218−0.018	−0.156	−0.367−0.054
Blue-collar worker					−0.241***	−0.331 to −0.151	−0.274***	−0.387 to −0.160	−0.265***	−0.379 to −0.152	−0.297***	−0.502 to −0.093
Behavior												
Physical activity at leisure times												
Activity that requires walking or cycling							−0.445***	−0.508 to −0.381	−0.441***	−0.504 to −0.378	−0.905***	−1.037 to −0.773
Participation in recreational sports							−0.784***	−0.864 to −0.703	−0.777***	−0.858 to −0.696	−1.365***	−1.528 to −1.202
Participation in hard training or sports competitions							−0.913***	−1.077 to −0.750	−0.908***	−1.071 to −0.745	−1.613***	−1.937 to −1.289
Neighborhood level												
Mean physical activity at leisure times									−0.891***	−1.330 to −0.452	−1.677***	−2.597 to −0.757
Mean physical activity at work									0.751***	0.558−0.945	0.897***	0.520−1.274
Living in rural area									−0.028	−0.124−0.068	0.014	−0.164−0.193
*Random effects*												
Variance components¹												
Neighborhood level	1.416		0.031		0.026		0.041 (0.202)		0.005 (0.073)		0.014 (0.119)	
ICC _neighborhood_ (%)	20.68		0.97		0.77		1.10		0.15		0.36	
*N*	33		33		33		33		33		33	
Individual level	11.490		11.280		11.055		10.986		10.969 (3.312)		12.173 (3.489)	
ICC _individual_ (%)	67.91		78.02		77.08		75.04		75.02		75.99	
*N*	25,604		25,604		17,738		17,561		17,561		12,990	
−2*loglikelihood	446,788.3 (df = 4)		411,924.6 (df = 14)		302,098.5 (df = 20)		230,982.0 (df = 22)		230,923.0 (df = 25)		96,228.11 (df = 25)	

*Note*: Model A includes all individuals who participated in Tromsø Studies more than twice; Model B, as a subgroup of Model A, includes individuals observed in the same neighborhood. SES: socioeconomic status; ICC: interclass correlation coefficient; CI: confidence interval; standard deviations (df) in parentheses.

* *p* < 0.1, **p < 0.05, ***p < 0.01.

¹Based on likelihood ratio test, neighborhood- and individual-level variances are significant at a 0.001 probability level.

## Results

### Descriptive characteristics

**Figure 2.**
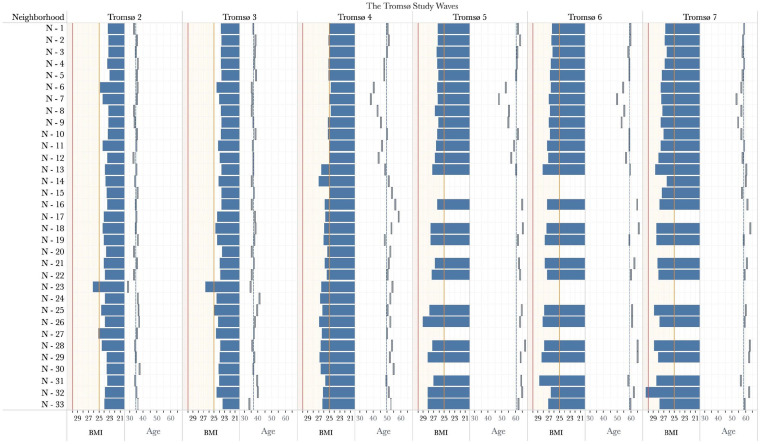
BMI and age variation in neighborhoods throughout the Tromsø Studies. We broke down the mean BMI and mean age for each neighborhood based on the Tromsø Study waves. For the panels illustrating mean BMI: neighborhoods are listed downwards on the y-axis, and each one represents the mean BMI in each Tromsø Study; the orange line refers to the overweight threshold (BMI = 25), and the red line refers to the obesity threshold (BMI = 30). The BMI panels show that many of the neighborhoods have mean BMIs that are lower than the overweight threshold in the Tromsø 4 and earlier studies. Nonetheless, after Tromsø 4, the average BMI in all neighborhoods exceeded the overweight threshold. For the panels showing mean ages: dashes display the mean age of the neighborhoods for each Tromsø Study, and the blue line indicates the wave’s mean age. The x-axis is between Tromsø 2 and Tromsø 7, and the y-axis limit is between 1 and 33 neighborhoods. Mean BMI at the neighborhood level ranged from 23.1 to 30.5. Throughout the Tromsø Studies, there were also differences in the mean BMI values of the neighborhoods.

Table I presents the characteristics of the neighborhood- and individual-level variables. Mean BMI increased constantly from Tromsø 2 to Tromsø 7, in line with the previous studies conducted in Tromsø [24]. We observed that this situation could raise the mean BMI limits from a normal weight to the overweight level. Furthermore, the average BMI of people living in the same neighborhood throughout the Tromsø Study surveys differed significantly from that of those living in different neighborhoods (Figure 2).

The mean age in Tromsø 2 was 35 years and, due to the sample panel structure, it steadily increased up to 61.15 years in Tromsø 7. Females accounted for 52% of our sample, but the rate of participation by gender varied between studies. The majority of the participants were married and living in an urban area. The variables related to SES indicate that the education level of participants changed from survey to survey; we also observed that the educational level of the participants’ mothers mostly fell within the low category (81% on average). Based on age, the number of unemployed or retired participants increased in Tromsø 7, while blue-collar workers decreased. Appendix A presents the descriptive characteristics of those included in Model B.

### Association between neighborhood health behaviors and body mass index in Northern Norway

Table II summarizes the coefficients and ICC values for both models via regression from the unconditional model to explanatory variables. The null model, Model A/1, showed that approximately 68% of BMI variation was at the individual level, and 21% occurred between neighborhoods. After controlling for sample demographics (Model A/2), we saw a quadratic relationship with age and found that BMI values were higher among middle-aged participants but lower among older and younger participants. In addition, individuals who reported their marital status as married/registered partnership and divorced/widow(-er)/separated were more likely to have a lower BMI (respectively, −0.219 kg/m², 95% confidence interval (CI) −0.282 to −0.156; −0.306 kg/m², 95% CI −0.385 to −0.228) than those who did not have a relationship or reported that they had never been married. Also, we found that the participants’ BMI increased significantly from Tromsø 3 to Tromsø 7 compared with Tromsø 1. Incorporating the SES variables (Model A/3) showed higher BMI among respondents reporting low education. Additionally, we showed that participants who reported higher maternal education had a lower BMI (−0.593 kg/m², 95% CI −0.862 to −0.323). Interestingly, maternal education level was more influential than both the moderate (−0.101 kg/m², 95% CI −0.159 to −0.043) and high education levels (−0.292 kg/m², 95% CI −0.373 to −0.211) of the participants. Also, being a blue-collar worker was significantly associated with a lower BMI than non-working people.

In Model A/4, the addition of the individual-level behavioral variable showed that those participants who were physically active during their leisure times had significantly lower BMI values (−0.913 kg/m², 95% CI −1.077 to −0.750). Furthermore, after controlling for physical activity during leisure time, the BMI association between white-collar workers and non-working individuals was in the same direction and significant (−0.114 kg/m², 95% CI −0.232 to −0.004); it was not at the same level as in the case of blue-collar workers. After accounting for all neighborhood-level variables, Model A showed that individual-level BMI was significantly associated with the overall health behaviors detected in the same neighborhood − in addition to the effects of individuals’ own physical activity. We found that BMI values (−0.891 kg/m², 95% CI −1.330 to −0.452) of participants were progressively lower for participants living in neighborhoods with more physically active populations. Another significant result was that individuals in neighborhoods where inhabitants were mostly employed in jobs requiring manual work, had a higher BMI on average (0.751 kg/m², 95% CI 0.558 to 0.945) than individuals in other neighborhoods. We found no evidence of an association between the urban or rural characteristics of neighborhoods, and the BMI values of their residents.

In Model B, we narrowed our sample group to individuals living in the same neighborhood. The Model B results shown in Table II are generally in line with our findings in Model A. However, when we focus on average leisure time physical activity level in the neighborhoods, the association of neighborhood health behavior was almost twice as high as the Model A results (−1.677 kg/m², 95% CI −2.597 to −0.757). We also saw the same double association on the individual-level variable of physical activity during leisure times, which varied from simple walking to strenuous training activities compared with a sedentary lifestyle. Under this model, taken independently, we found no significant evidence of a BMI association between white-collar workers and non-working individuals.

## Discussion

In this study, we examined the association between the average leisure time physical activity level of the neighborhood and BMI in a comprehensive longitudinal sample of adult individuals from Northern Norway. The most important finding that emerged from this study was that the overall leisure time physical activity level of the neighborhood was associated with the individual-level BMI values in the same neighborhood. This finding is consistent with Durlauf’s explanation of imitation behavior, assuming that an individual will find a particular behavior comparatively more desirable when evaluating alternative behavioral choices if others have previously behaved or are currently behaving the same way [7]. In addition, as Sund et al. point out, some characteristics of the neighborhood’s social or physical environment may also be relevant in weight gain [11].

The second significant finding was that people who live in neighborhoods where people work in labor-intensive jobs have higher BMI values than others, regardless of labor activity. Sund et al.’s finding that physically demanding workers gain more weight than others at the neighborhood level but not at the individual level [11] is supported by our results. As they discussed, this is attributable to manual workers’ reduced leisure time physical activity and their less desirable dietary habits outside of the workplace [11]. Furthermore, education is important in relation to gaining awareness about poor diets and sedentary lifestyles that can have an adverse impact on BMI [11,25]. Furthermore, we showed that a mother’s level of education affected an individual’s BMI, with stronger effects observed in individuals with higher maternal education.

Although some studies show that the neighborhood is significantly associated with BMI [8,9], other studies conclude that environmental conditions do not affect BMI [4]. The former studies draw on the idea that the characteristics of residential areas can promote salutogenic or pathogenic health behaviors of their residents through exposure to factors such as area-level unemployment and access to social and physical resources such as food, healthcare, and green space [26]. For example, a study conducted in the USA found that people living in less deprived areas were more physically active since those areas had enough facilities available to allow for physical activities [10]. Moreover, residents of areas with a higher density of fast-food outlets have lower rates of fruit and vegetable consumption, and food desert and obesogenic environments increase excessive food intake [25].

Our study includes both strengths and limitations. One of the advantages is the availability of data from a large, well-characterized cohort of individuals for an extended period. The relationship between BMI and obesity and overweight is well established in the literature [4,8,11,13]. Anthropometric measurements such as waist circumference are also used in obesity studies [14,27]. Since BMI is the most useful measure of overweight and obesity at the population level, we used BMI and calculated it using WHO-recommended height and weight measurements [13]. Because of the large sample size and data availability, we were able to investigate the effects of neighborhoods. By correctly assigning individuals to these neighborhoods both in time and geography, we were able to generate a continuous contextual exposure from Tromsø 2 to 7, which covers 37 years. Other studies have also emphasized the significance of understanding the many levels in a population, noting that ignoring one level will alter estimates at other levels [11,19].

The major limitation of this study was that our neighborhood level consists of data from specific years of the Tromsø Studies. During the gap years, individuals were likely to move between/within neighborhoods or outside of the Tromsø municipality, and there were no available records of their residential areas in the Tromsø Study. Since it was possible that movers who relocated within the Tromsø municipality either relocated to areas that resembled their previous neighborhoods or to neighborhoods with higher/lower SES [28], we might have missed the influence of these movers in our results. Considering this possibility, we repeated our analysis only for the individuals who were observed in the same neighborhood to provide a stable sample for estimating neighborhood effect (see Model B) [4,11]. The dataset in our study, on the other hand, was made up of people who lived in a single county. As a result, even after accounting for rural and urban differences, the variability in the physical environment was limited. In addition, individuals may tend to overstate leisure time physical activity levels, which may have introduced bias and diluted its relationship with BMI [14]; nonetheless, the relationship between self-reported leisure time physical activity level and chronic diseases is well established [27]. According to Rödjer et al., self-reported leisure time physical activity level is associated with the presence of several cardiovascular risk factors [27]. Finally, it is worth noting that some of the participants were likely to be members of the same biological family, and genetic information, as well as family environment characteristics, may have an impact on weight gain [29].

Apart from neighborhood effects being academically intriguing, its concept has been utilized by policymakers for reducing the potential of adverse neighborhood effects [30]. Our findings indicated that individuals’ BMI differed in the context of a neighborhood, implying that regional policymakers should consider not only the physical or socioeconomic conditions of the neighborhood but also how to improve the overall health behaviors of the people who live there. As ﻿ Sundquist et al. [31] stated, multidisciplinary interactions between health researchers, city planners, economists, and policymakers are required in neighborhood-level strategies; governmental initiatives should incorporate multidimensional correlates of physical activity.

Further studies regarding the role of relocation over time would be worthwhile [11]. Thus, it could be investigated whether individuals choose the neighborhood in which they live because of their health behaviors or whether their health behaviors are affected by overall health behaviors in the area in which they live.

## Conclusion

The present study was designed to determine the relationship between the overall leisure time physical activity level of the neighborhood and the BMI of its residents in a large 37-year panel sample of adults. Taken together, the results suggested that there was an association between neighborhood-level variables and residents’ BMIs. Specifically, having neighbors who were physically active during their leisure time was associated with lower BMIs.
